# A Vaginal Angiomyofibroblastoma as a Rare Cause of a Prolapsing Vaginal Mass: A Case Report and Review of the Literature

**DOI:** 10.1155/2018/8579026

**Published:** 2018-04-29

**Authors:** Harriet Calvert, Supuni Kapurubandara, Yogesh Nikam, Raghwa Sharma, Anita Achan

**Affiliations:** ^1^Department of Obstetrics and Gynaecology, The Maitland Hospital, Maitland, NSW, Australia; ^2^Department of Obstetrics and Gynaecology, John Hunter Hospital, Newcastle, NSW, Australia; ^3^Department of Obstetrics and Gynaecology, Westmead Hospital, Sydney, NSW, Australia; ^4^Sydney West Advanced Pelvic Surgery (SWAPS), Sydney, NSW, Australia; ^5^University of Sydney, Sydney, NSW, Australia; ^6^Department of Tissue Pathology and Diagnostic Oncology, Institute of Clinical Pathology and Medical Research, Westmead Hospital, Sydney, NSW, Australia; ^7^Western Sydney University, Sydney, NSW, Australia

## Abstract

**Introduction:**

Angiomyofibroblastoma (AMFB) is a rare, benign, mesenchymal cell tumour which presents as a slow-growing mass. It is most commonly seen in the vulva and is often mistaken for Bartholin's abscess. It is histologically diagnosed by the presence of stromal cells intermingled with small blood vessels. It is morphologically similar to cellular angiofibroma and aggressive angiomyxoma, the latter of which is locally invasive and has a possibility of metastasis and a high risk of local recurrence. There is one reported case of an AMFB undergoing sarcomatous transformation.

**Case Report:**

We report a case of a multiparous, 36-year-old woman with an anterior vaginal mass which was inappropriately treated as a vaginal prolapse prior to definitive surgical management. This is only the second reported case of an AMFB presenting as a prolapsing mass.

## 1. Introduction

Angiomyofibroblastoma (AMFB) is a rare, benign, mesenchymal tumour that most commonly occurs as a slow-growing mass in the vulva, first described in 1992 [1]. It is often misdiagnosed as Bartholin's gland cyst [1, 2].

This type of solid tumour has also less commonly been described in the vagina and the inguinoscrotal region of men.

It is most prevalent in women in the reproductive age group with a mean age of 45 and varies in size (from 0.5 to 23 cm) but is usually less than 5 cm [1–4].

Histologically, it has a defined border and is characterised by alternating hypo- and hypercellular areas with numerous blood vessels [1, 5].

## 2. Case Report

We describe a case of a 36-year-old multiparous (G3P2) woman who presented with an acute episode of pelvic pain. She was referred to a general gynaecological clinic after ultrasound findings revealed a 4.1 cm complex left ovarian cyst suggestive of an endometrioma.

She also reported a 2-year history of a bulge that protruded from her vagina and was associated with discomfort and dyspareunia and occasionally required digital reduction especially with tampon use. She had been diagnosed with a vaginal prolapse by a gynaecology clinic at another institution.

Her past medical history consisted of migraines with aura, exercise induced asthma, and a family history of breast cancer (half-sister). She had never had a PAP smear.

On bimanual examination, a well-delineated solid mass was found on the anterior vaginal wall in the midline, measuring 5 cm by 5 cm. There was no evidence of pelvic organ prolapse with good support of the uterus, posterior wall, and anterior wall above the mass. The cervix was visualised anteriorly and there was no evidence of cervical excitation. A routine PAP smear was performed with difficulty secondary to the vaginal mass.

With respect to investigations, Ca 125 was 29 U/mL giving a low relative malignancy index. A repeat ultrasound scan demonstrated a 2.9 cm left ovarian cyst, suggestive of an endometrioma and a solid mass inferior to the uterus and anterior to the vagina, displacing the bladder (Figure 2).

**Figure 1:**
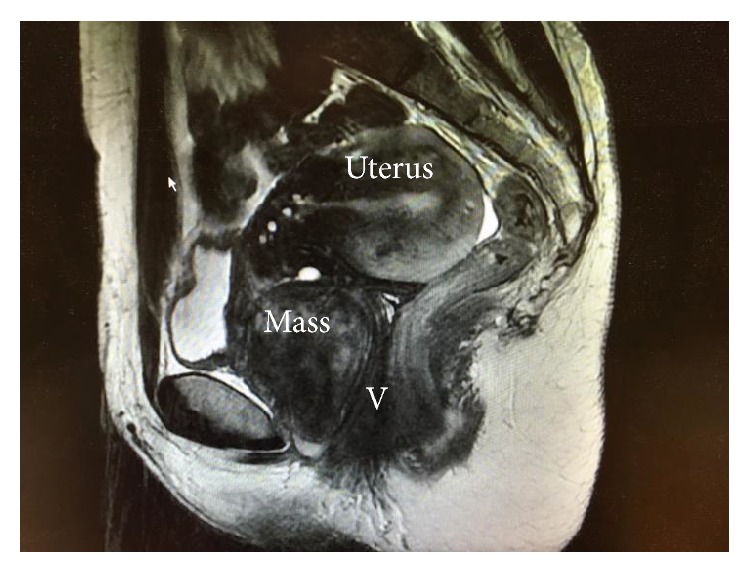
Sagittal section of MRI showing the 45 mm × 50 mm solid mass in the vesicovaginal septum in relation to the uterus, mass, and vagina (V).

**Figure 2:**
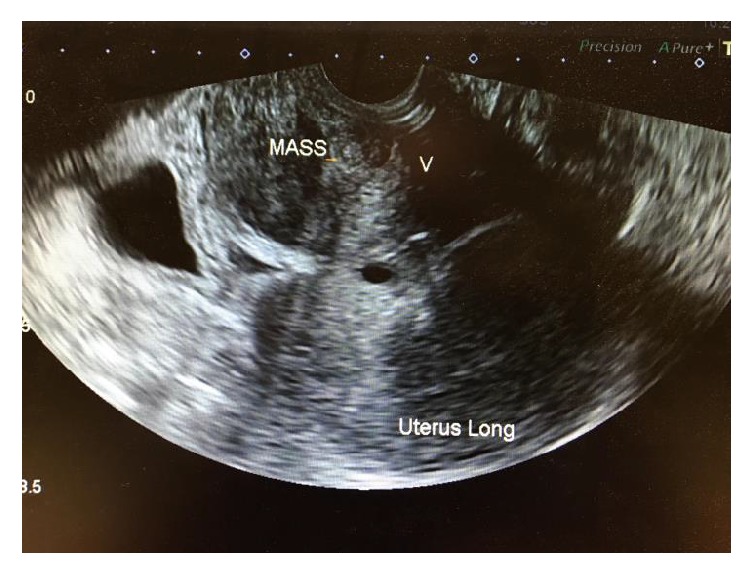
Ultrasound scan showing relation of mass to uterus and vagina.

On Magnetic Resonance Imaging, a 45 mm × 50 mm solid mass in the vesicovaginal septum with a well-defined margin was demonstrated (Figure 1). The mass was displacing the bladder anteriorly and displacing the urethra towards the left of the midline. T2 imaging showed a predominantly hypointense, heterogenous signal with areas of hyperintensity. There was mild enhancement after gadolinium injection. Close to the external urethral orifice, the interface between the mass and the urethra was ill defined. Evidence of a left ovarian endometrioma and endometriosis deposits were seen elsewhere in the pelvis.

These MRI findings suggested that the mass was either endometriosis with surrounding reactive fibrous and smooth muscle proliferation, neoplasm, or an infection relating to a urethral diverticulum. After a multidisciplinary meeting with a urogynaecologist, the patient underwent an examination under anaesthesia, diagnostic laparoscopy, cystoscopy, excision of endometriosis, and excision of the vaginal mass.

**Figure 3:**
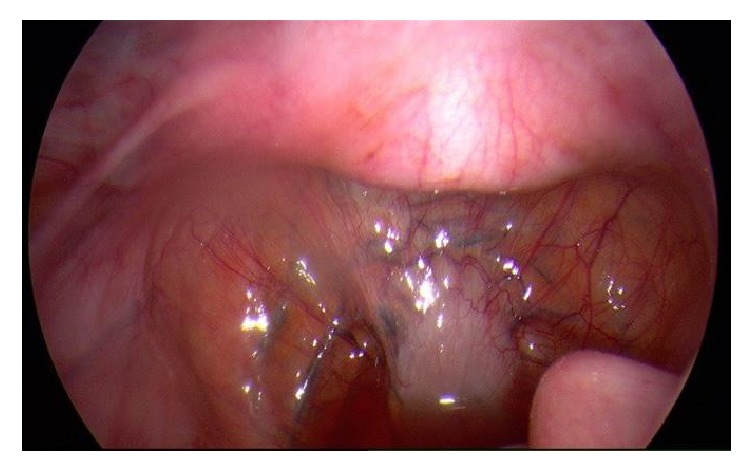
View of mass on diagnostic laparoscopy; uterus anteverted.

**Figure 4:**
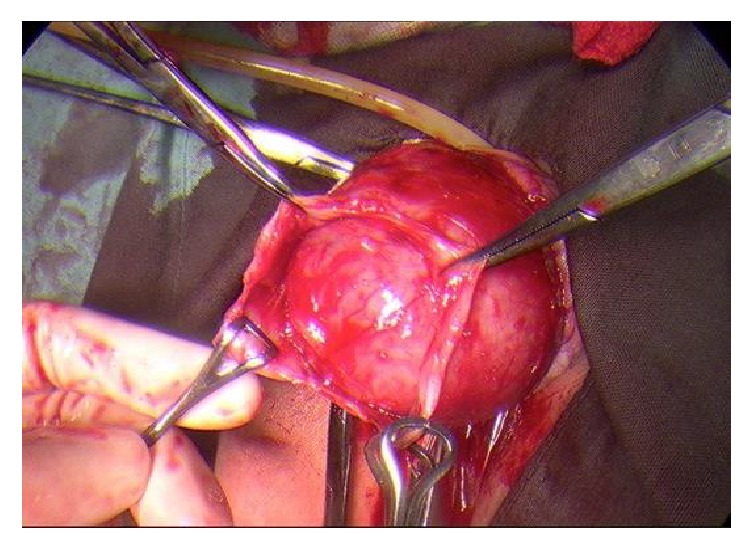
Marsupialisation of vaginal mass from anterior vaginal wall.

**Figure 5:**
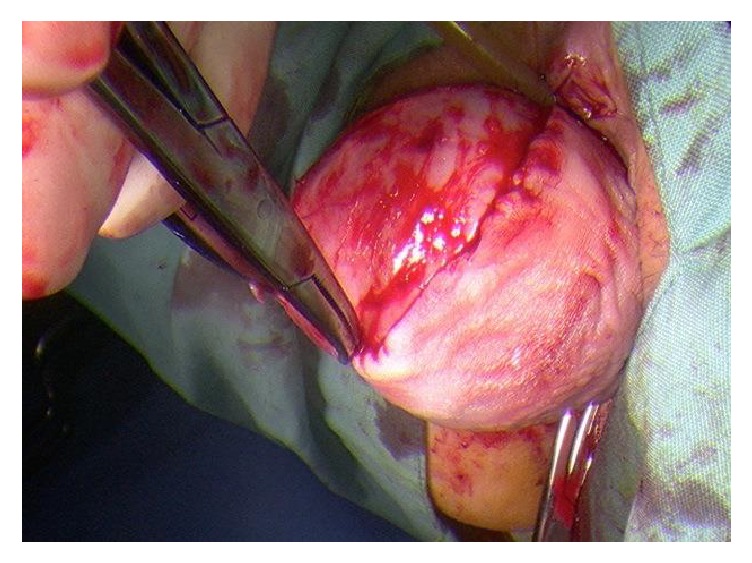
Excision of vaginal mass from anterior vaginal wall.

**Figure 6:**
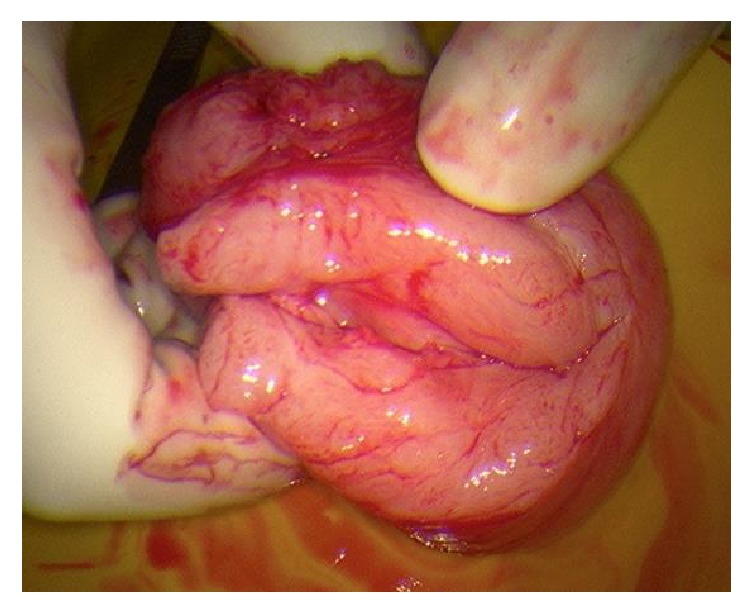
Cut surface of mass.

The vaginal mass was removed with laparoscopic assessment via a midline incision on the anterior vaginal wall with lateral dissection around the cystic structure (Figures 3–6). A cystoscopy and urethroscopy suggested no involvement and the cyst was enucleated. Multiple haemostatic sutures were needed with surgical snow to achieve haemostasis and the defect was closed. A repeat cystoscopy and urethroscopy showed no injury.

**Figure 7:**
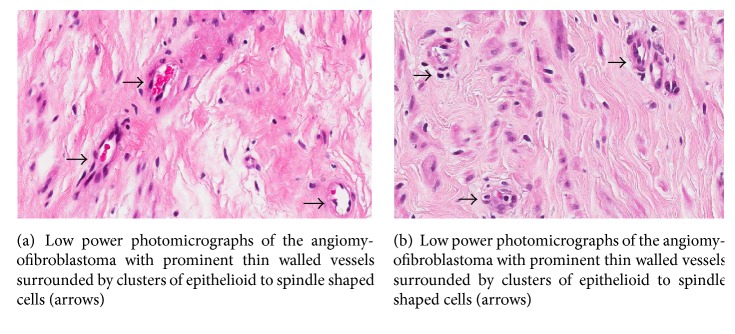


**Figure 8:**
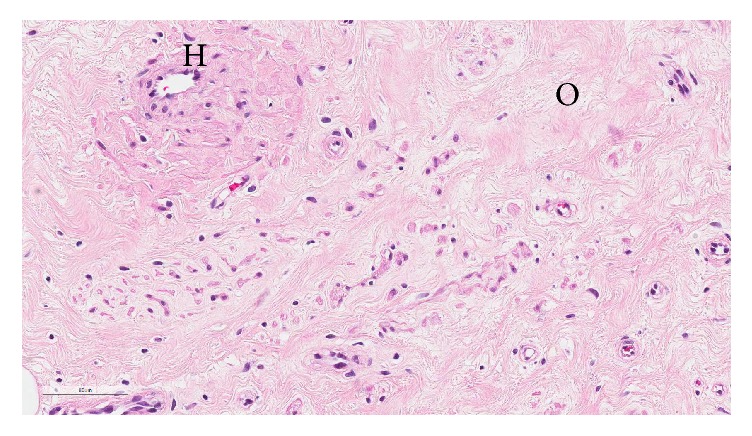
Cross section showing alternating hypercellular areas around blood vessels (H) and hypocellular (O) areas containing slender collagen fibrils with no evidence of necrosis.

Histopathological macroscopic assessment of the mass showed pale tan tissue surrounded by a thin capsule and on sectioning a homogeneous whorled tan tissue (Figure 6). Microscopically the low power photomicrographs showed a well-circumscribed border. It comprised collagenised areas of epithelioid to spindled cells with small to thin walled arborizing vessels. Aggregation of cells around vessels was noted and there were no atypical mitoses, necrosis, or atypia (Figures 7 and 8).

The immunohistochemistry showed positive desmin, SMA, CD34, and vimentin. The cells displayed high intensity nuclear positivity for progesterone and oestrogen receptors. These findings were consistent with a diagnosis of angiomyofibroblastoma.

## 3. Discussion

AMFB is a very rare benign, mesenchymal tumour with less than 100 cases previously having been reported in the literature. There has been a reported age range of 17–86 with a mean age at presentation of 45 [2, 6, 7].

It commonly presents as a painless, slow-growing, vulval mass and is most commonly diagnosed as Bartholin's cyst or abscess (46%) or a lipoma (15%) [2]. There is only one other reported case of it presenting as a prolapsing vaginal mass [8]. There is often a delay in diagnosis with a mean duration of 29 months between initial symptoms and diagnosis [2, 6].

AMFB is morphologically similar to other invasive mesenchymal cell tumours such as aggressive angiomyxoma (AAM) and cellular angiofibroma and they share many overlapping immunohistochemical and structural features [9, 10].

It is diagnostically challenging to differentiate between AMFB and AAM but important due to the latter's locally invasive nature, the possibility of metastasis, and the high risk of local recurrence [11, 12]. AMFB can be diagnosed by a higher cellularity, distinct border, plump stromal cells, increased presence of small blood vessels, and a lesser degree of stromal myxoid change [6]. Other differential diagnoses include cellular angiofibroma and vulvovaginal myofibroblastoma. Cellular angiofibromas are uniformly cellular with thick-walled, hyalinised blood vessels without surrounding aggregation of epithelioid or plasmacytoid cells. Adipocytes are often found in the periphery [10]. Vulvovaginal myofibroblastomas characteristically contain ovoid, spindle, or stellate cells in a variety of architectural patterns. They also do not have the perivascular aggregates seen in AMFB [10]. Both cellular angiofibromas and myofibroblastomas exhibit the loss of RB1 and FOXO1A1 genes due to the deletion of the 13q14 chromosomal region. This typical loss of genetic material is not found in AMFB [13].

Immunohistologically, AMFB tumours have been found to be strongly positive for vimentin, positive for desmin, and to a lesser degree alpha-smooth muscle actin. Staining is rarely useful in differentiating between tumour types [13]. The stromal cells are characteristically positive for oestrogen and progesterone receptors, suggesting a hormonal role in the development of the tumour [14].

There have only been 5 previous reports of MRI findings of an AMFB. All report a mass with well-defined margins and as in our case they have been found to appear as a heterogeneous signal intensity on T2-weighted MRI. All other cases reported fast and persistent enhancement on dynamic gadolinium-enhanced MRI whereas ours showed only mild enhancement [15, 16].

The other studies found a mass with homogeneous intermediate signal intensity on T2 weighted MRI [7, 17, 18].

Ultrasound has been reported to be useful in assessing heterogeneity, vascularity, and delineating infiltration and relation to surrounding structures [7, 19].

It is widely accepted that AMFB can be treated with wide local excision with clear margins. There has only been one case report of a benign local recurrence. This was a pedunculated mass 5 × 3 cm arising from the vaginal vault which was excised with clear margins. Upon follow-up 14 months later 3 small, nodular growths were found close to the site of excision on the anterior and posterior vaginal walls which, when excised, showed the same features as the previous tumour with no transformation [8].

There has also been reported one case of previously diagnosed AMFB undergoing sarcomatous change. A 13 cm vulval mass was resected which showed many accepted features of an AMFB however did show focal sarcomatous change at the resected margin. At 2 years, the mass had recurred at the same site and resection demonstrated a 14 cm mass comprised of only the high-grade sarcomatous component with vascular invasion that was not previously present [20].

Another reported case of a locally invasive recurrence of AMFB at 2 years after resection was due to a misdiagnosed AAM on the original specimen [21]. The local recurrence rate of AAM after clear margin resection has been reported to be up to 47% [22–25].

## 4. Conclusion

The majority of AMFB occur in the vulva, most commonly presenting as a painless mass.

Vaginal AMFB are rarer and may present later with dyspareunia, awareness of a vaginal mass, or an incidental finding on exam [26–28]. Wide local excision is the recommended treatment, with enough surrounding tissue to enable the pathologist to differentiate between AMFB and the locally infiltrative AAM.

MRI and US can be useful imaging modalities depending on location of the tumour. Due to the rarity of cases, there are no recommendations on long-term monitoring but due to the reported instances of tumour recurrence and sarcomatous transformation we suggest that follow-up should be considered until at least 2 years postoperatively [8, 20].
